# Mammalian‐Like Inflammatory and Pro‐Resolving Oxylipins in Marine Algae

**DOI:** 10.1002/cbic.202000178

**Published:** 2020-05-05

**Authors:** Hans Jagusch, Tim U. H. Baumeister, Georg Pohnert

**Affiliations:** ^1^ Department of Instrumental Analytics/Bioorganic Analytics Institute for Inorganic and Analytical Chemistry Friedrich Schiller University Jena Lessingstraße 8 07743 Jena Germany; ^2^ Fellow Group Plankton Community Interaction Max Planck Institute for Chemical Ecology Hans-Knöll-Straße 8 07745 Jena Germany

**Keywords:** biosynthesis, inflammation, natural products, oxylipins, pro-resolving

## Abstract

Oxylipins constitute a family of oxidized fatty acids, that are well known as tissue hormones in mammals. They contribute to inflammation and its resolution. The major classes of these lipid mediators are inflammatory prostaglandins (PGs) and leukotrienes (LTs) as well as pro‐resolving resolvins (Rvs). Understanding their biosynthetic pathways and modes of action is important for anti‐inflammatory interventions. Besides mammals, marine algae also biosynthesize mammalian‐like oxylipins and thus offer new opportunities for oxylipin research. They provide prolific sources for these compounds and offer unique opportunities to study alternative biosynthetic pathways to the well‐known lipid mediators. Herein, we discuss recent findings on the biosynthesis of oxylipins in mammals and algae including an alternative pathway to prostaglandin E_2_, a novel pathway to a precursor of leukotriene B_4_, and the production of resolvins in algae. We evaluate the pharmacological potential of the algal metabolites with implications in health and disease.

## Graphical abstract

**The uniqueness of oxylipin** endowment in mammals is cracked by marine algae. They also form bioactive lipid mediators such as prostaglandins (PGs), leukotrienes (LTs), and resolvins (Rvs) in response to wounding as is observed in mammals. Contributions to the algal oxylipin chemistry kicked off the discovery of novel biosynthetic pathways in mammals with implications in health and disease.

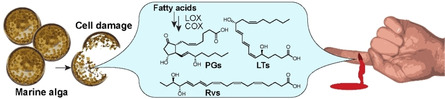

## Introduction

The finding that certain bioactive natural products are produced in unrelated organisms opens opportunities for the investigation of their biosynthesis, for solving supply issues, and for the elucidation of their multifunctionality. But also mining for novel structures derived from similar biosynthetic pathways in different producers is a successful strategy for natural product research. In particular, the field of oxylipins was stimulated by the reciprocal investigation of mammalian, plant and algal pathways. Oxylipins are oxidized derivatives of unsaturated fatty acids that function as versatile tissue hormones in mammals but also as central hormones in plants. In algae they serve mainly as chemical defense.

Eicosanoids that are derived from unsaturated C_20_ fatty acids are termed lipid mediators since they regulate the initiation and resolution of inflammation in mammals.^1^, ^2^ The main families of these immunomodulatory compounds are the pro‐inflammatory prostaglandins (PGs), the leukotrienes (LTs), and the pro‐resolving resolvins (Rvs). They are biosynthesized by enzymes such as cyclooxygenases (COXs) and/or lipoxygenases (LOXs) and mediate biological effects via cognate G protein‐coupled receptors (GPCRs).^1^, ^3^ These potent molecules provide targets for the development of anti‐inflammatory drugs such as COX‐inhibitory coxibs and nonsteroidal anti‐inflammatory drugs (NSAIDs) to intervene in the pathogenesis of inflammation.^4^ However, side effects and inefficiency of these drugs are incentives to search for better alternatives. Resolvin derivatives have recently been recognized as promising anti‐inflammatory drug candidates due to high biological activity.^5^ Their medical use is yet constrained due to the limited supply caused by low yielding sources for the natural product and lack of economic, efficient syntheses.^6^

**Figure 1:**
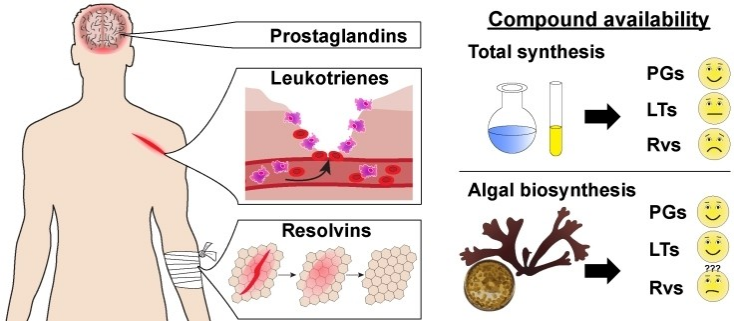
Inflammatory and pro‐resolving lipid mediators in humans as inspiration for the development of anti‐inflammatory drugs. Compound availability issues are complicating their pharmaceutical application. Algae could provide opportunities to overcome this hurdle.

Marine algae produce structurally diverse bioactive oxylipins derived from C_20_ fatty acids as well and could thus be a valuable alternative source for lipid mediators. Algal oxylipins have mainly been examined in the context of their chemical ecology; their pharmaceutical potential has not yet been comprehensively evaluated.^7^ Herein, we discuss novel oxylipins from marine algae with structural similarities or identity to lipid mediators in mammals, comprising the classes of prostaglandins, leukotrienes, and resolvins. We focus on own recent contributions to algal oxylipin chemistry with special emphasis on biosynthetic aspects and implications in health and disease. The concept is strengthened that natural products occurring in different kingdoms of life provide unique opportunities for biosynthetic research and offer new avenues in the development of pharmaceutical applications (Figure 1).

### Oxylipins in Algae

Oxygenated lipids are widespread in micro‐ and macroalgae with universal structural elements, but species‐specific structures.^8^ In particular, microalgae belonging to the group of diatoms as well as brown and red macroalgae are capable of producing high amounts of C_20_ polyunsaturated fatty acids (PUFAs) such as arachidonic acid and eicosapentaenoic acid. These fatty acids are the precursors for mammalian‐type oxylipins. PUFAs are processed by LOXs, COXs, and cytochrome P450 (CYPs) and subsequently transformed by hydroperoxide isomerases, hydroperoxide lyases, epoxide hydrolases, and peroxide reductases.^9^, ^10^, ^11^, ^12^ LOX activity with varied regioselectivity has been reported for marine diatoms, brown algae, and red algae.^13^, ^14^, ^15^, ^16^ The initially formed hydroperoxides give rise to a structurally diverse family of algal oxylipins.

In algae, a hormonal function of oxylipins is still under debate, but there is clear evidence that these compounds serve as pheromones that support sexual reproduction and act as chemical defense against pathogens and grazers.^17^, ^18^, ^19^ Often, the biosynthesis of algal oxylipins is induced upon wounding of the cells or tissue, leading to a rapid conversion of the stored precursor lipids to give the free fatty acids that function as substrates for further down‐stream metabolism.^20^ Regulation of oxylipin biosynthesis shares elements with that in mammals, where the enzymatic machinery (LOXs, COXs) increases fatty acid conversion to lipid mediators upon tissue damage‐induced rise of intracellular calcium.^3^

Algae are a rich source for hydroxylated eicosapentaenoic acids (HEPEs) or tetraenoic acids (HETEs), polyunsaturated aldehydes (PUAs), fatty acid hydroperoxides, and epoxy alcohols.^7^ Oxylipin formation has been particularly well studied in the red algae *Gracilaria chilensis* and *Gracilaria vermiculophylla* where a lipase‐, LOX‐ or COX‐mediated conversion of galacto‐ and phospholipids gives rise to eicosanoids.^21^ Among *Gracilaria* oxylipins, 7,8‐dihydroxy‐ETE (7,8‐diHETE) acts as a natural antifouling agent and deterrent against grazers.^21^, ^22^ In contrast to their prevalence in macroalgae, arachidonic acid‐derived HETEs are not the most abundant hydroxylated oxylipins in microalgae. In the diatom *Skeletonema costatum*, for example, eicosapentaenoic acid‐derived HEPEs prevail including 15‐hydroxy‐EPE (15‐HEPE) that inhibits the growth of the algicidal bacterium *Kordia algicida*.^23^ Diatoms are also well‐known producers of short‐chain α,β,γ,δ‐polyunsaturated aldehydes (PUAs). PUA mediated chemical defense is activated in response to cell damage and causes an increase in the offspring mortality of diatom‐grazing copepods.^24^ Fatty acid hydroperoxides, such as 15‐hydroperoxy‐eicosapentaenoic acid as well as epoxy alcohols formed by the diatoms *Chaetoceros socialis* and *Thalassiosira rotula* also contribute to this toxicity.^8^, ^25^

### Prostaglandins

Most described eicosanoids from algae are LOX‐derived, but also COX‐cyclized lipid mediators with high structural resemblance to mammalian hormones are observed. We focus here on prostaglandins with activity in algae and mammals. PGF_2α_ and PGE_2_ are the most common and best‐evaluated prostaglandins in algae. Both are derived from arachidonic acid and function primarily as defense molecules for macroalgae, while their function in microalgae is unknown.^26^, ^27^, ^28^, ^29^ PGF_2α_ and PGE_2_ content can be associated with invasiveness of macroalgae as reported for the spreading of the red seaweed *G. vermiculophylla* in the Northern Atlantic.^30^ Algal PGE_2_ has also been linked to occasionally occurring food poisoning (e. g., nausea, vomiting, diarrhea) upon ingestion of raw red algae from the edible genus *Gracilaria*.^31^

**Figure 2:**
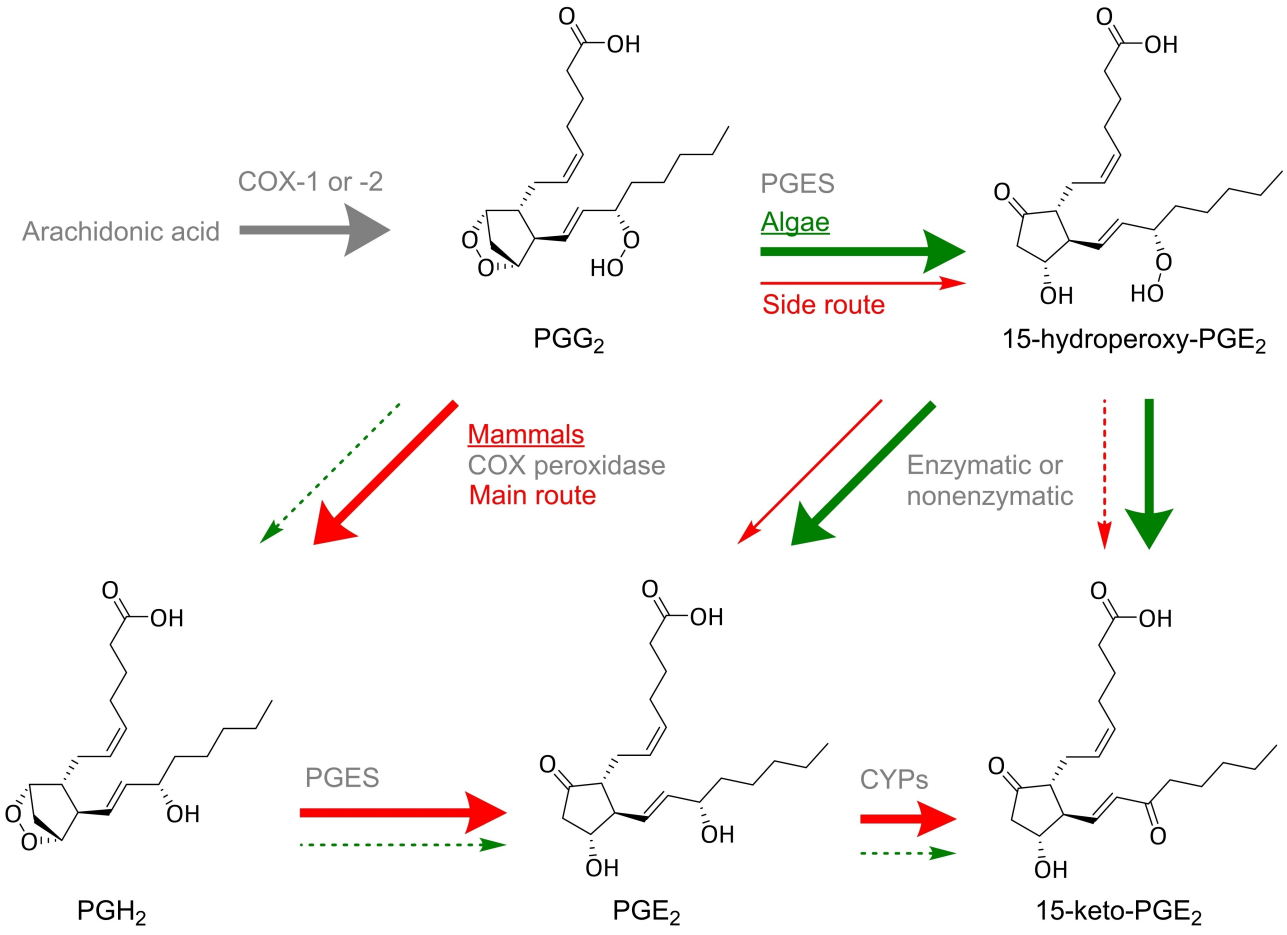
Biosynthesis of PGE_2_ in algae and in mammals. The canonical pathway in mammals follows the thick red arrows. Arachidonic acid is cyclized by COX‐1 or COX‐2 to PGG_2_, which is reduced by the peroxidase activity of the COX to PGH_2_ and subsequently converted by prostaglandin E synthase to PGE_2_. An alternative pathway was discovered in the red alga *Gracilaria vermiculophylla* (thick green arrows). There PGG_2_ is transformed into the novel intermediate 15‐hydroperoxy‐PGE_2_, which is enzymatically or nonenzymatically transformed to PGE_2_. Upon re‐investigation of the biosynthesis in human macrophages, the alternative route via 15‐hydroperoxy‐PGE_2_ was established in mammals as well (thin red arrows).^32^

Mining of *G. vermiculophylla* for arachidonic acid‐derived oxylipins with emphasis on prostaglandins revealed 15‐hydroperoxy‐PGE_2_. This hydroperoxide is the biosynthetic intermediate to PGE_2_ and 15‐keto‐PGE_2_ in algae (Figure 2).^32^ Interestingly, the hydroperoxide was previously not observed in mammals where the biosynthesis via PGH_2_ is well established. It becomes clear that mammals and algae employ different major routes to the same prostaglandin (Figure 2). The availability of the algal 15‐hydroperoxy‐PGE_2_ allowed screening the human lipidome for this metabolite. Indeed, human macrophages, key producers of lipid mediators during inflammation, contain this hydroperoxide.^32^, ^33^ 15‐Hydroperoxy‐PGE_2_ is in fact a hitherto unidentified intermediate in an alternative route to PGE_2_ in mammals, where the formation of PGE_2_ proceeds along two independent pathways (Figure 2).^31^ The route via 15‐hydroperoxy‐PGE_2_ dominates in algae and renders a mixture of PGE_2_ and 15‐keto‐PGE_2_ whereas human macrophages preferentially rely on the canonical pathway via PGH_2_ with the 15‐hydroperoxy‐PGE_2_ route as a minor pathway (Figure 2).^32^

The discovery of an independent route via the hydroperoxide might provide a new paradigm for treating inflammation‐related diseases. Selective COX peroxidase inhibitors may be developed to alleviate carcinogenic effects from side‐products formed by the COX peroxide reductase domain while still allowing PGE_2_ biosynthesis to occur via the hydroperoxide pathway.^32^ Regarding ingestion of raw alga, 15‐hydroperoxy‐PGE_2_ might be converted to PGE_2_ inside the human stomach promoting food poisoning.

### Leukotrienes

Another class of mammalian‐like oxylipins in algae are arachidonic acid‐derived leukotrienes that function as immunomodulators in mammals. Leukotriene B_4_ (LTB_4_) is the most potent compound in mammals regulating inflammation and mediating chemotaxis of leukocytes for the host‐protective response.^34^ Elevated concentrations of LTB_4_ can lead to gastrointestinal toxicity.^35^ Only a few studies reported leukotrienes in marine algae. The two red macroalgae *G. vermiculophylla* and *Murrayella periclados* form LTB_4_ and derivatives with unknown biological function for the producers.^36^, ^37^

**Figure 3:**
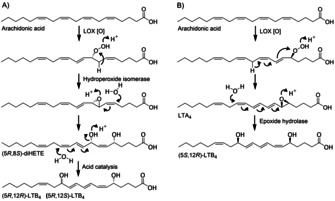
A) Proposed pathway for the formation of LTB_4_ isomers in algae. Arachidonic acid is oxygenated by an 8*R*‐LOX to yield a hydroperoxy‐fatty acid. This intermediate is then cyclized to the epoxide by a hydroperoxide isomerase followed by stereoselective enzymatic addition of water giving rise to (5*R*,8*S*)‐diHETE. Under acidic conditions, this precursor rearranges to (5*R*,12*R*/*S*)‐LTB_4_ (*ee*=95 %). B) Mammalian biosynthesis of (5*S*,12*R*)‐LTB_4_. Arachidonic acid is initially oxygenated by a 5*S*‐LOX forming 5‐HPETE that is subsequently dehydrated resulting in the precursor epoxide LTA_4_. This is then enzymatically hydrolyzed to LTB_4_.^38^, ^39^

Upon re‐investigation of oxylipins formed upon wounding by *G. vermiculophylla*, we identified a novel oxylipin, (5*R*,8*S*)‐dihydroxy‐ETE (5*R*,8*S*‐diHETE).^38^ This acid‐labile molecule rearranges via an 1,8‐diol‐forming mechanism to inflammatory LTB_4_ isomers as an enantiomeric mixture (Figure 3).^38^ This step is likely to occur abiotically rather than being enzymatically catalyzed. The algal metabolites exhibit an enantio‐specific potency toward the LTB_4_ receptor 1.^38^ LTB_4_ is likely generated in algae via 7,8‐epoxy‐eicosatetraenoic acid as illustrated in Figure 3A. This process might be initiated by an 8‐LOX/hydroperoxide isomerase sequence followed by a nonselective acid‐catalyzed dehydration/hydration reaction.^38^ This route differs significantly from the biosynthesis in mammals that is initiated by a 5‐LOX leading to 5,6‐epoxy‐eicosatetraenoic acid and LTB_4_ (Figure 3B).^38^, ^39^

The availability of LTB_4_ isomers enabled the investigation of structural requirements for receptor‐ligand interaction in mammals. The LTB_4_ receptor 1 is enantioselective with specificity for (5*s*,12*r*)‐LTB_4_.^38^ In addition to PGE_2_, algal leukotrienes and related hydroxylated fatty acids might also cause food poisoning.

### Resolvins

**Figure 4:**
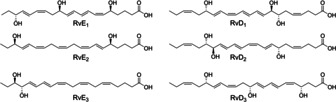
Selection of resolvins derived from eicosapentaenoic acid (E series) and docosahexaenoic acid (D series).

Prostaglandins and leukotrienes have been well known for decades and contribute mainly to inflammatory processes. In contrast, the relatively newly discovered class of specialized pro‐resolving mediators is involved in the resolution of inflammation. The concentration of the specialized pro‐resolving mediators, resolvins, lipoxins, protectins, and maresins is elevated upon inflammation.^40^ Resolvins are mainly derived from ω‐3‐PUFAs such as eicosapentaenoic acid, docosapentaenoic acid, and docosahexaenoic acid. Their formation is catalyzed by regioselective LOXs, COXs, and CYPs.^40^ These lipid mediators exhibit pro‐resolving effects including inhibition of pro‐inflammatory transcription factors, modification of the fatty acid composition of phospholipids, and activation of anti‐inflammatory transcription factors.^41^ Resolvins (Figure 4) are thus of interest for the development of pro‐resolving agents. Structures derived from those oxylipins are currently tested in pre‐clinical trials such as the RvE_1_‐analogue RX‐10045 for the treatment of keratoconjunctivitis sicca.^42^

Microalgae produce high amounts ω‐3‐PUFAs and harbor a diverse family of eicosapentaenoic acid‐converting LOXs.^43^, ^44^ They are therefore promising candidates for the production of resolvins. In fact, an initial survey revealed the diatoms *Coscinodiscus granii* and *Chaetoceros didymus* producing RvE_2_ and RvE_3_.^45^ These findings may lay the foundation for an aquaculture‐based supply of resolvins and other SPMs.

### Mechanisms of Enzymatic Transformations in Oxylipin Formation

**Figure 5:**
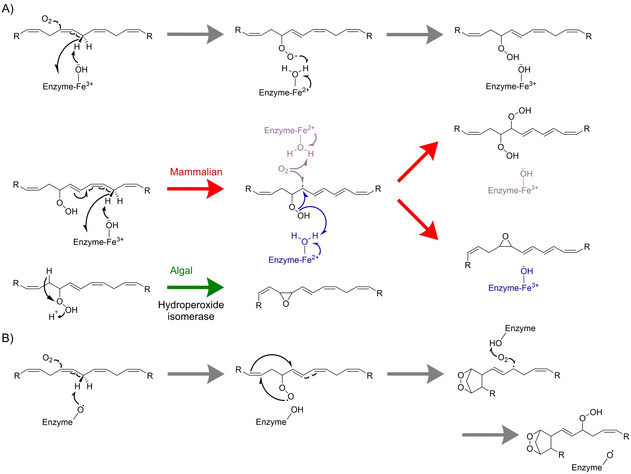
Central enzymatic steps in the biosynthesis of C_20_‐oxylipins in algae and mammals. A) LOX‐mediated oxidation with subsequent reaction steps catalyzed either by LOXs (mammals, red arrows) with different mechanisms (purple or blue) or by hydroperoxide isomerases (algae, green arrows). B) COX‐catalyzed cyclization in algae and mammals.

While the central classes of enzymes involved in the initiation of the biosynthesis of prostaglandins, leukotrienes, and resolvins in algae and mammals are similar, pronounced differences are observed in the downstream transformation of intermediate hydroperoxides. In algae as well as in mammals the multistep enzymatic conversion of fatty acids is triggered by calcium‐dependent sphingomyelinases, galactolipases, or phospholipases that release precursor fatty acids such as arachidonic acid, eicosapentaenoic acid, docosapentanoic acid, and docosahexaenoic acid.^3^, ^21^, ^46^ Subsequent oxygenation is mediated by LOXs and COXs according to Figure 5.^46^, ^47^, ^48^ These iron‐containing enzymes are substrate‐specific and stereoselective. The resulting intermediates can be further metabolized by a second catalytic domain of a bi‐functional enzyme, such as a peroxide reductase activity of LOXs and COXs. Alternatively, other enzymes such as epoxide hydrolases that are prevalent in mammals or hydroperoxide isomerases that are algae‐specific can transform the reactive intermediates.^47^, ^48^, ^49^, ^50^

Phylogenetic analysis of LOXs from plants, mammals, moss, corals, algae and cyanobacteria suggests a common origin and also determinant amino acids and widely conserved reaction mechanisms.^51^ In a first, rate‐limiting step, LOXs abstract one hydrogen from a bisallylic methylene carbon forming a conjugated radical that is then oxygenated to a peroxy radical followed by a LOX‐mediated reduction to the corresponding anion and subsequent protonation yielding a hydroperoxide (Figure 5A).^52^ LOX‐derived hydroperoxy fatty acids are processed differently in algae and mammals (Figure 5A). In algae, the hydroperoxide is either reduced by the lipoxygenase itself to give a hydroxy‐fatty acid or cyclized to an epoxide via a hydroperoxide isomerase.^49^, ^53^ The epoxide can be opened by stereoselective addition of water yielding dihydroxy‐fatty acids such as (5*R*,8*S*)‐diHETE.^38^, ^53^ Micro‐ and macroalgae have also pronounced hydroperoxide lyase activities releasing polyunsaturated aldehydes as chemical defense.^12^ Here untypical transformations are observed including the action of halolyases introducing chlorine into the fatty acid metabolites.^54^

Mammalian LOXs can reduce the hydroperoxy intermediate forming a hydroxy‐fatty acid as also observed in algae.^55^ Alternatively, as it is the case in the LTB_4_ biosynthesis, LOXs can catalyze a second abstraction of a bisallylic hydrogen and mediate epoxide formation.^55^ Epoxides can be further hydrolyzed via epoxide hydrolases yielding dihydroxy‐fatty acids such as LTB_4_.^50^ 1,2‐Diols are also accessible via the LOX‐mediated introduction of a second hydroperoxy group (Figure 5A) with subsequent double peroxide reduction assembling a 1,2‐diol.^55^

Alternatively, a COX‐mediated cyclization and second dioxygenation with protonation of the peroxy radical generating an endoperoxide/hydroperoxide intermediate is observed (Figure 5B).^47^, ^56^ How this intermediate is metabolized differs between algae and mammals. In mammals, the endoperoxide/hydroperoxide is reduced by a COX as reported for the transformation of PGG_2_ into PGH_2_.^57^ The endoperoxide motif can then be further processed to various end products such as PGE_2_ catalyzed by the prostaglandin E synthase.^58^ The peroxide reduction step mediated by a COX‐type activity in algae is not very prominent. The amino acid sequence of the algal enzyme shares just about 20 % of the mammalian counterpart, giving a possible explanation for this low activity.^9^, ^32^ Instead of being reduced, as it is the case in mammals the endoperoxide/hydroperoxide intermediate is likely processed by an enzyme opening the endoperoxide followed by reduction to PGE_2_.^32^

The general enzyme classes transforming unsaturated fatty acids in mammals and algae are thus very similar. Their organization in biosynthetic pathways are however fundamentally different, leading in diverse routes to identical products or to the formation of structural or enantiomeric isomers.

## Conclusion

Oxylipins are not solely biosynthesized in mammals where they function as versatile inflammatory and pro‐resolving lipid mediators but are also generated in algae. Algae produce these metabolites in alternative pathways compared to mammals using different enzymes and intermediates but rely on similar transformation principles. Based on biosynthetic considerations derived from investigations of algae, novel mammalian pathways were discovered, and novel isomers of lipid mediators are accessible. Algae therefore provide important resources for pharmaceutical tools and guide novel strategies for intervention in oxylipin biosynthesis in mammals. Poor availability of mammalian lipid mediators has yet constrained potential pharmaceutical and research applications. This problem may be overcome by algae producing such mammalian‐like lipid mediators.

## Conflict of interest

The authors declare no conflict of interest.
